# Effect of Sowing Date on the Development of Lacy Phacelia (*Phacelia Tanacetifolia* Benth.)

**DOI:** 10.3390/plants11223177

**Published:** 2022-11-21

**Authors:** Zuzana Kubíková, Hana Smejkalová, Helena Hutyrová, Antonín Kintl, Jakub Elbl

**Affiliations:** 1 Agricultural Research, Ltd., Zahradní 1, 664 41 Troubsko, Czech Republic; 2 Department of Agrosystems and Bioclimatology, Faculty of AgriSciences, Mendel University in Brno, Zemědělská 1, 613 00 Brno, Czech Republic

**Keywords:** phacelia, sowing date, growth and height intensity, weed infestation, yield parameters

## Abstract

Experiments with lacy phacelia (*Phacelia tanacetifolia* Benth.) were carried out in the period 2017–2021, during which the effect of sowing date on the stand development was assessed (height, coverage, accomplishment of individual growth stages, and weed infestation). It was demonstrated that the sowing date affects the stand growth and development. In earlier sowing dates, plants developed slower than in later sowing dates but reached a greater average height (926 mm). The lowest height (802 mm) was exhibited by plants from May (sowing 3). The third sowing date also showed the worst stand canopy closure and at BBCH 31, the stand canopy closure in Sowing 1, 2, and 3 was 82.3%, 77.8%, and 67%, respectively. The third sowing date was also the highest weed infestation. The effect of sowing date on yield and yield parameters was also monitored (weight of a thousand of grains and germinative capacity). Based on the measured data, it can be concluded that the influence on the weight of thousands of grains was minimal and in germinative capacity. Significant differences were recorded only in first year in which the germinative capacity of plants from the later sowing dates was reduced.

## 1. Introduction

Sustainable agriculture postulates the protection of biodiversity and puts emphasis on balanced cropping practices that are able to fulfil all expected functions such as the maintenance or enhancement of the quality of cultivated soils, prevention of the occurrence of harmful agents in the grown crops, and maintenance of yield stability of agricultural production. One of possible strategies is using inter-crops that are currently coming to the fore. Lacy phacelia (*Phacelia tanacetifolia* Benth., PHCTA) is a popular intercrop [1].

Lacy phacelia originates in the Pacific areas of North America, but it was spread in Europe by the 19th century. It is grown mainly as an inter-crop [2,3] that is reaching high yields of green matter [4]. It can be used both for green fertilization and as a fodder [5,6]. The only closely related crop is *Phacelia congesta* Hook. (Blue curls) [7] and is therefore an excellent disrupter of crop rotation. It can be used in mixtures for intermediate belts in orchards and vineyards [8] as well as in various erosion-control systems. It is not a host plant of sugar beet nematode [9], does not make possible its reproduction, and according to some sources can even reduce its populations [10].

Lacy phacelia is also an important honey plant [11,12], producing nectar and pollen of high quality; it is highly attractive not only for bees but also for other insect species, not only pollinators [13,14,15,16] but also other beneficial insects, including predators and parasitoids.

Lacy phacelia is drought-resistant [17] and grows well even in precipitations of ca. 200–500 mm per year. It prefers lighter, properly aerated soils with a good supply of nutrients. Optimum pH ranges from 6.6 to 8.5. Compacted, waterlogged, or acidic soils are inappropriate. It is relatively tolerant to cold, beginning to emerge in a temperature of about 3 °C. Young plants can withstand frosts of on average up to −8 °C. In tropics and subtropical areas, it can be grown as a winter crop [18,19], but in colder regions it usually freezes out [20]. The species has modest requirements for cultivation, does not suffer either from diseases or pests, and is strong enough to compete weeds [21,22,23]. As an intercrop, it can reduce the occurrence of weeds in the main crop [2]. A basic precondition for the establishment of high-quality stand with good competitiveness is high-quality seed. The influence of the date of sowing on plant development was studied, for example in winter wheat [24], where there was a reduction in grain yield with later sowing date [25]. The influence of sowing date on growth and development of agricultural crops was also studied for soybeans [26] and cotton [27]. The effect of sowing date on the vegetation period was described by Bowie et al. [18], Thrasyvoulou and Tsirakoglou [28], or Lermi and Palta [29]. The mentioned authors found that the sowing date has a significant effect on the height of the plant, the beginning of flowering, and the length of the vegetation period of the agricultural crops.

In conditions of central Europe (CR), seed stands of lacy phacelia are established in spring. The goal of this study was to verify a zero hypothesis that the sowing date has no influence on the rate of development, plant height, coverage, and yield parameters, and to evaluate the effect of spring sowing date of seed stands on the growth and development of lacy phacelia, competitiveness and weed infestation, and on the yield and quality of obtained seeds.

## 2. Results

**Figure 1:**
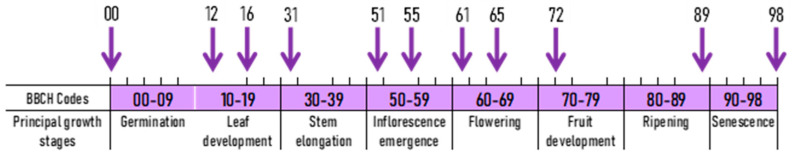
Illustration of the growth stages in which lacy phacelia plants were evaluated. BBCH 00—sowing; BBCH 12–98—ten terms of measurements (height—evaluated in all terms; coverage of phacelia—evaluated in terms BBCH 10, 12, 16, 31, 51, 61, and 72; coverage of weeds and number of plants per m^2^ for the lacy phacelia and for individual weed species were evaluated in BBBCH 61).

Variability and interactions between measured values were analyzed using ANOVA (Table 1). The main sources of variability were the following factors: (a) year, (b) sowing date, and (c) development stage of phacelia (Figure 1). The effects of these factors were described by ANOVA analysis at the level of significance *p* < 0.05, *p* < 0.01 and *p* < 0.001. Based on these data, the effect of year and developmental stage can be considered equally strong (Table 1). The influence of the year was given by the different course of weather in individual years of experiment (2018–2020). In particular, the year 2018 was extremely warm and dry, which influenced the height, coverage, and speed of development of the phacelia. The influence of developmental stage on selected parameters was also evident (Table 1). Different height, growth intensity, coverage, and daily coverage increase were found in the individual development phases (Table 1; Figure 1). Above all, the influence of the interactions of some factors was found for the monitored parameters. For example, the factors year and sowing significantly influenced plant height and plant coverage. In contrast, no interactions were detected in the number of days for reaching the respective developmental stages.

### 2.1. Rate of Growth and Development, Vegetation Period

The dates when the lacy phacelia from three sowing dates (1st and 2nd half of April and May) reached the selected developmental stages were recorded during three years. Differences were observed between the individual sowing dates in the rate of growth and development.

There were no significant differences in the rate of growth in the first growth phases (Figure 1). In Sowing 1 (1st half of April), emergence was recorded after 7–14 days; in Sowing 2 (2nd half of April) and Sowing 3 (May), it was after 6–13 days and after 6–10 days, respectively. Therefore, the hypothesis H0 that the sowing date does not influence the rate of emergence and initial development of plants was not rejected.

**Figure 2:**
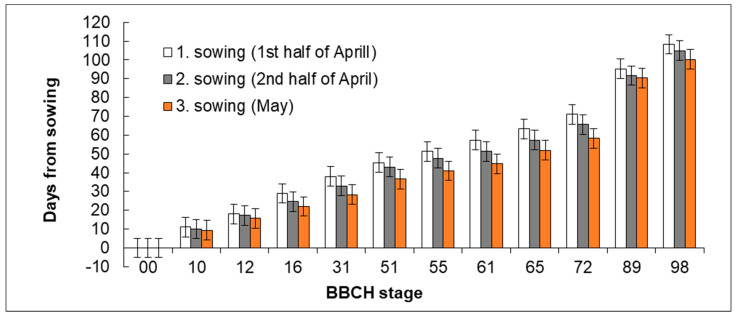
Effect of sowing date on the number of days for reaching the respective developmental stages. Mean values (*n* = 9) of individual variants are shown for the specific developmental stage (BBCH) in three years of study ± SD. Different letter indices are present only in variants in which significant differences were found (*p* < 0.05). If no letter indices are included, no significant difference was found between the respective variants.

**Figure 3:**
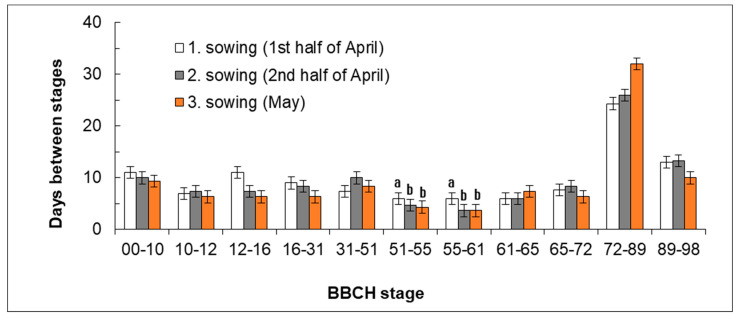
Effect of sowing date on the number of days for reaching the range of developmental stages. Mean values (*n* = 9) of individual variants are shown for the specific range of developmental stages (BBCH) in three years of study ± SD. Different letter indices are present only in variants in which significant differences were found (*p* < 0.05). If no letter indices are included, no significant difference was found between the respective variants.

A similar trend of gradually accelerating development was shown also in the development of leaves (BBCH 12–16), which was slower in Sowing 1 than in Sowing 2 (2nd half of April) and Sowing 3 (May, Figure 2). However, the trend was not statistically significant. In the period of elongation, the rate of development accelerated in Sowing 1 (1st half of April). The period of elongation falls to the second third of May in Sowing 1 (1st half of April), which is usually connected with the arrival of more intensive rainfalls. In contrast, the beginning of spring is usually dry. Combined with the increasing temperatures, the rainfalls supported growth and stem elongation in the plants from Sowing 1 (1st half of April). In Sowing 2 (2nd half of April), leaves were developing and stem elongation started in the second third of May whereas plants from Sowing date 3 (May) were just emerging in that period. The flower bud formation was significantly slowest in Sowing 1 (1st half of April). The significant differences between Sowing 1 (1st half of April) and others sowing dates were recorded between BBCH stages 51 (beginning of inflorescence emergence) and 61 (beginning of flowering). Plants from Sowing 3 (May) exhibited the most rapid development until the period of seed formation, then it slowed down. In contrast, the period of ripening was the longest one of all sowing dates (Figure 3).

### 2.2. Height and Growth Intensity

**Figure 4:**
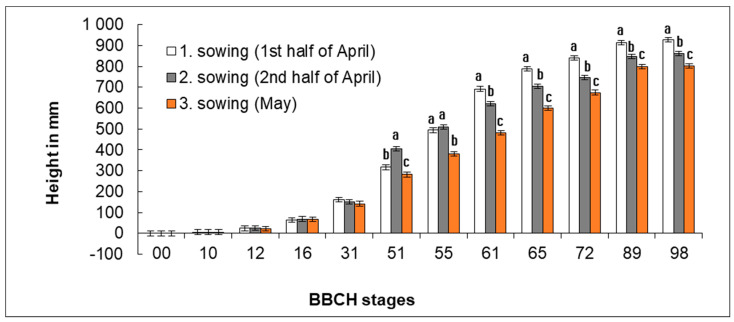
Effect of sowing date of the height of plants reached in the respective developmental stages. Average values (*n* = 90) of individual variants are shown for the specific developmental stages (BBCH) in three years of study ± SD. Different letter indices are added only to variants in which significant differences were found (*p* < 0.05). If there are no letter indices, no significant differences between the individual variants were found.

Stand height was recorded in the studied developmental stages (Figure 4). Daily height increments (Figure 4) were calculated from the recorded height values and time difference between the reach of growth stages for individual time periods between the following developmental stages.

Significant differences (*p* < 0.05) in height were found in stands from different sowing dates in different developmental stages from the growth stage BBCH 51 between individual sowing rates (Figure 4). The significantly greatest final height was reached by plants in Sowing 1 (1st half of April) where an average height of plants ranged, depending on the year, from 754 to 1069 mm. In Sowing 2 (2nd half of April), the final height of plants was on average by 64 mm smaller than in Sowing 1 (1st half of April) and ranged from 703 to 953 mm. Plants from Sowing 3 (May) showed the smallest height, being on average by 127 mm smaller than plants from Sowing 1 (1st half of April). Differences between individual variants in final height of plants were significant. The average height of plants from Sowing 3 (May) was 681–925 mm.

The smallest height non-significant differences between the respective sowing dates were observed in the initial growth stages up to BBCH 31. In BBCH 51, the greatest height was reached by plants from Sowing 2 (2nd half of April), whose average height ranged from 287 to 493 mm. Plants from Sowing 1 (1st half of April) reached a height that was on average by 89 mm smaller than that of plants from Sowing 2 (2nd half of April). Plants from Sowing 3 (May) were smallest in height and a height difference was on average 35 mm as compared with Sowing 1 (1st half of April). In the following growth stages, the growth of plants from Sowing 2 (2nd half of April) slowed down, and in BBCH 61, plants with the greatest height were already those from Sowing 1 (1st half of April). In this stage, plants from Sowing 1 (1st half of April) reached an average height of 498–830 mm. Plants from Sowing 2 (2nd half of April) were on average by 72 mm smaller than those from Sowing 1 (1st half of April). Plants from Sowing 3 (May) were on average by 211 mm smaller than those from Sowing 1 (1st half of April), their average height being 357–571 mm. Height differences in the later developmental stages (BBCH 51–98) were statistically significant (*p* < 0.05).

**Figure 5:**
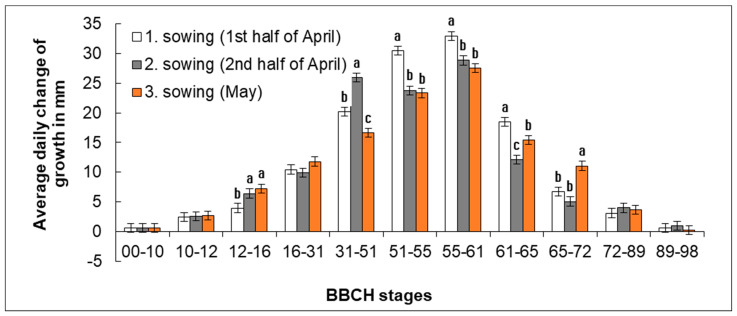
Effect of sowing date on average daily height increment reached between the respective developmental stages BBCH. Average values (*n* = 90) of individual variants are shown for the specific range of BBCH developmental stages in three years of study ± SD. Different letter indices are added only to variants in which significant differences were found (*p* < 0.05). If there are no letter indices, no significant differences were found between the individual variants.

Intensity of plant growth changed depending on the growth stage and sowing date (Figure 5). At the beginning of development, i.e., up to BBCH 10–12, the growth of plants was slow, and its intensity was nearly identical in all sowing dates, without significant differences. The growth rate gradually increased from 0.6 mm (BBCH 00–10) per day up to 2.5–2.8 mm per day (BBCH 10–12). First significant differences (*p* < 0.05) between the sowing dates started to show in the period of developing true leaves (BBCH 12–16) and the slowest growth was recorded in plants from Sowing 1 (1st half of April), which was significantly slower than in plants from Sowing 2 and Sowing 3. In this period, the growth intensity in plants from Sowing 1 was on average 4.0 mm per day. In plants from Sowing 2 (2nd half of April) and Sowing 3 (May), it was 6.5 mm and 7.3 mm per day, respectively. Then, the growth intensity began to increase rapidly in plants from Sowing 1 (1st half of April) and the highest growth intensity in plants from this sowing date was reached in the period of bud formation (BBCH 51–61) when height increments ranged on average from 30.4 to 32.9 mm per day. In this period of development, the intensity of elongation growth was significantly higher in plants from Sowing 1 (1st half of April) than in those from Sowing 2 (2nd half of April) and Sowing 3 (May). In plants from Sowing 2 (2nd half of April), the highest growth intensity was recorded from the beginning of elongation to the beginning of flowering (BBCH 31–61) and ranged from 25.9 to 28.8 mm per day. From the beginning of elongation up to the beginning of bud formation (BBCH 31–51), the growth intensity of plants from Sowing 2 was significantly higher than in plants from Sowing 1 (1st half of April) and Sowing 3 (May). Plants from Sowing 3 (May) exhibited the highest growth intensity in the stage of bud formation (BBCH 51–61), which—similarly as in plants from Sowing 1 (1st half of April)—ranged from 23.8 to 27.8 mm per day. The growth intensity began to slow down rapidly in the period of flowering. The slowest growth was recorded in plants from Sowing 3 (May). In the following vegetation phases (since BBCH 72–89), no significant differences were found. It follows from the measured data that the H0 hypothesis was rejected; the sowing date affected the growth intensity.

### 2.3. Coverage and Intensity of Stand Canopy Closure

**Figure 6:**
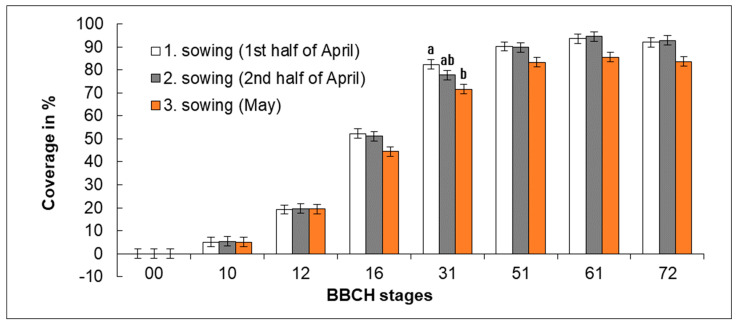
Effect of sowing date on the coverage of plants reached in individual developmental stages. Average values (*n* = 9) of individual variants are shown for the specific developmental stages (BBCH) in three years of study ± SD. Different letter indices are added only to variants in which significant differences were found (*p* < 0.05).

**Figure 7:**
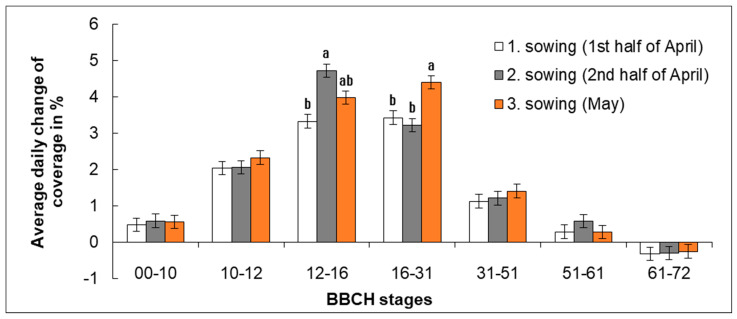
Effect of sowing date on average daily increment of coverage reached between individual BBCH developmental stages. Average values (*n* = 9) of individual variants are shown for the specific range of BBCH developmental stages in three years of study ± SD. Different letter indices are added only to variants in which significant differences were found (*p* < 0.05). If there are no letter indices, no significant differences were found between the individual variants.

Another studied parameter was coverage (Figure 6) and rate of stand canopy closure (Figure 7). Daily increments of coverage for individual time periods between the subsequent developmental stages were calculated from the recorded values of coverage and time differences between the reach of respective growth stages.

During the development of phacelia, the coverage increased very intensively, and an effect of the sowing date was observed. Stands from Sowing 1 and Sowing 2 exhibited canopies that were better closed. The stand from Sowing 3 featured a generally worse canopy closure. Differences in stand canopy closure were statistically significant between Sowing 1 (1st half of April) and Sowing 3 (May) only in BBCH 31 (see Figure 6), when leaf coverage in Sowing 1 (1st half of April), Sowing 2 (2nd half of April), and Sowing 3 (May) was from 76.7 to 86.0%, from 73.3 to 83.3%, and from 65.0 to 81.7%, respectively. In all three sowing dates, the stands of lacy phacelia reached a maximum canopy closure in the period of flowering. Later, the leaves gradually began to dry, the vegetation was coming to an end and the coverage was decreasing. In the period of flowering (BBCH 61), phacelia plants from Sowing 1 (1st half of April) exhibited leaf coverage from 91.7 to 97.7% per plot in dependence on year. In Sowing 2 (2nd half of April) and Sowing 3 (May) it was from 86.7 to 98.7% and from 79.7 to 97.0%, respectively. No statistically significant differences in canopy closure were recorded between Sowing 1 (1st half of April) and Sowing 2 (2nd half of April).

The canopy closure of lacy phacelia stands was relatively fast. The most intensive stand canopy closing occurred in the period of leaf development from BBCH 12 to BBCH 31. In BBCH 12, the average stand canopy closure was 19.2% in Sowing 1, 19.6% in Sowing 2 (2nd half of April), and 18% in Sowing 3 (May). In BBCH 16, the average canopy closure in Sowing 1, 2, and 3 was 52.2%, 51.1%, and 40%, respectively. At the beginning of elongation (BBCH 31), the stand canopy closure in Sowing 1 (1st half of April), 2 (2nd half of April), and 3 (May) was 82.3%, 77.8%, and 67%, respectively.

The lowest rate of stand canopy closing was recorded in Sowing 1 where it was from 3.3% (BBCH 12–16) to 3.4% (BBCH 16–31) of leaf coverage per day in the period of leaf formation. Slightly accelerated canopy closing occurred in the later stage of leaf formation. The highest rate of canopy closing was observed in Sowing 2 (2nd half of April) in the period of initial leaf formation (BBCH 12–16), when it reached on average 4.7% of leaf coverage per day; then it slowed down to 3.2% in the later stage of leaf formation (BBCH 16–31). In Sowing 3 (May), the highest rate of stand canopy closing was from 3.4% (BBCH 12–16) to 4.6% (BBCH 16–31), with a lower rate being recorded during the earlier stage of leaf formation; later, the intensity of canopy closing increased (only up to BBCH 31). The intensity of stand canopy closing is shown in Figure 7. The H0 hypothesis that the date of sowing does not affect the stand canopy closure was disproven.

### 2.4. Number of Plants per Square Meter and Weed Infestation

**Figure A1:**
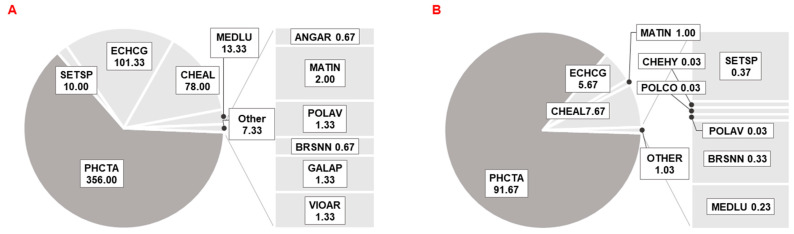
Number of plants per m^2^—Sowing 1 (12th of April) in 2018 (**A**) and leaf coverage in %—Sowing 1 (12th of April) in 2018 (**B**).

**Figure A2:**
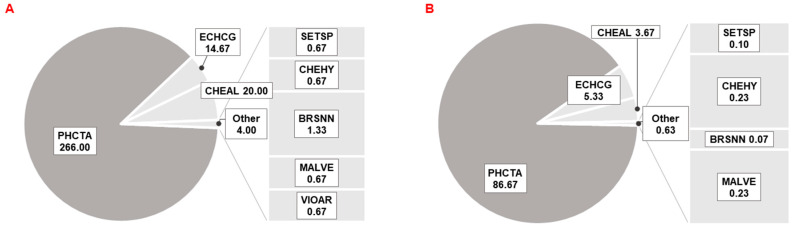
Number of plants per m^2^—Sowing 2 (24th of April) in 2018 (**A**) and leaf coverage in %—Sowing 2 (24th of April) in 2018 (**B**).

**Figure A3:**
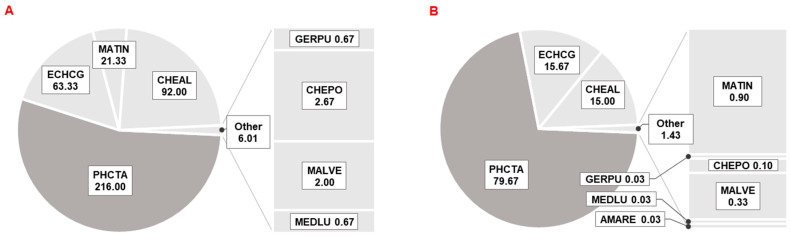
Number of plants per m^2^—Sowing 3 (10th of May) in 2018 (**A**) and leaf coverage in %—Sowing 3 (10th of May) in 2018 (**B**).

**Figure A4:**
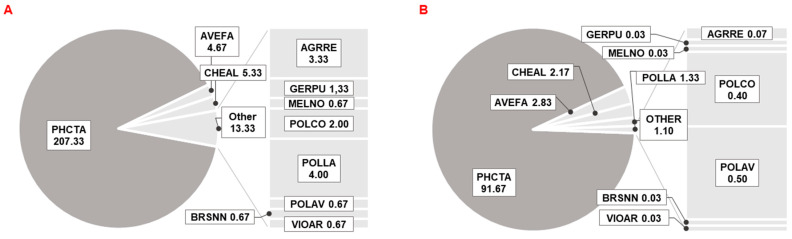
Number of plants per m^2^—Sowing 1 (5th of April) in 2019 (**A**) and leaf coverage in %—Sowing 1 (5th of April) in 2019 (**B**).

**Figure A5:**
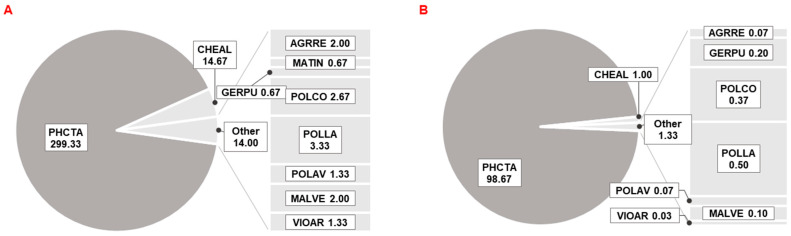
Number of plants per m^2^—Sowing 2 (16th of April) in 2019 (**A**) and leaf coverage in %—Sowing 2 (16th of April) in 2019 (**B**).

**Figure A6:**
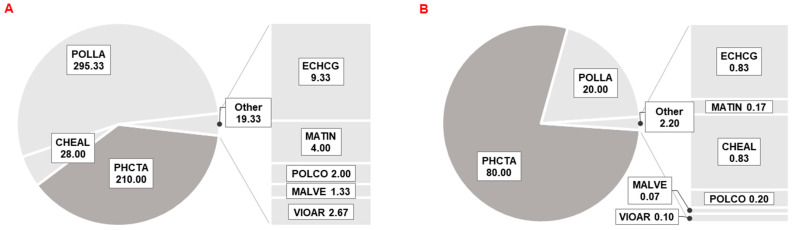
Number of plants per m^2^—Sowing 3 (7th of May) in 2019 (**A**) and leaf coverage in %—Sowing 3 (7th of May) in 2019 (**B**).

**Figure A7:**
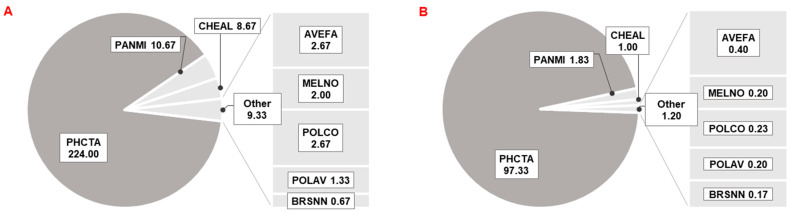
Number of plants per m^2^—Sowing 1 (8th of April) in 2020 (**A**) and leaf coverage in %—Sowing 1 (8th of April) in 2020 (**B**).

**Figure A8:**
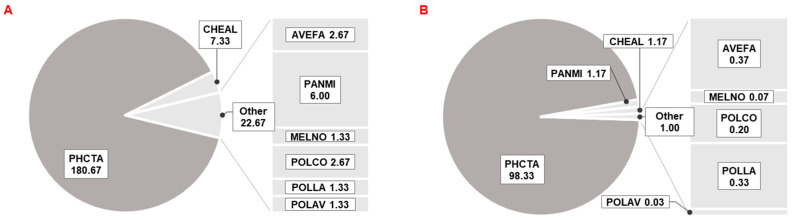
Number of plants per m^2^—Sowing 2 (22nd of April) in 2020 (**A**) and leaf coverage in %—Sowing 2 (22nd of April) in 2020 (**B**).

**Figure A9:**
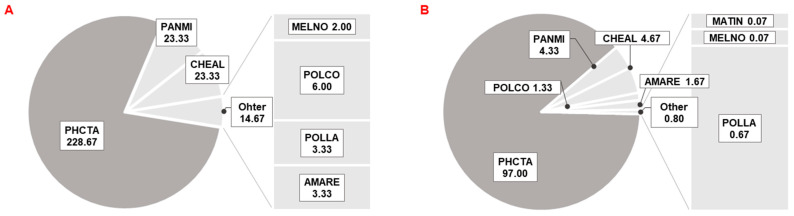
Number of plants per m^2^—Sowing 3 (7th of May) in 2020 (**A**) and leaf coverage in %—Sowing 3 (7th of May) in 2020 (**B**).

At the beginning of flowering, the number of plants per square meter was evaluated both in phacelia and in the accompanying flora (weeds) together with the coverage of individual weed species. Differences were observed both in the overall weed infestation, and in the species composition of weeds, both being affected by the sowing date and year. The species composition of weeds for the respective sowing dates of lacy phacelia and years as well as their numbers and coverage as compared with the lacy phacelia are presented in Appendix A, Figure A1, Figure A2, Figure A3, Figure A4, Figure A5, Figure A6, Figure A7, Figure A8 and Figure A9. A total number of monocotyledonous weed species recorded in the experiments was 5: common wild oat (*Avena fatua* L., AVEFA), couch grass (*Elytrigia repens* (L.) Desv. Ex Nevski, AGRRE), cockspur (*Echinochloa crus-galli* (L.) O. Beauv., ECHCG), yellow bristle grass (*Setaria pumila* (Poir.) Roem & Schult., SETPU), and common millet (*Panicum miliaceum* L., PANMI). The most frequently occurring monocotyledonous weed was cockspur, which was represented in all sowings in the warm year of 2018, mostly in the stands from Sowing 3 (May). In 2019, it occurred only in Sowing 3 (May), and in Sowing 1 (1st half of April), there was common wild oat which also occurred in 2020 in Sowing 1 (1st half of April) and Sowing 2 (2nd half of April). The common millet occurred only in 2020; it was a crumble from the intercrop when a mixture was sown on the plot with the common millet which formed viable seeds thanks to the warm autumn. The massive occurrence of common millet probably suppressed the cockspur that did not occur in that year. Yellow bristle grass and couch grass showed only minority representation.

The composition of dicotyledonous weeds was more colorful. There were altogether 16 species of dicotyledonous weeds. In terms of measured values, the most frequently occurring weed was white goosefoot (*Chenopodium album* L., CHEAL) which occurred in all years in all sowing dates. Also sporadically occurring were other goosefoot species such as maple-leaved goosefoot (*Chenopodiastrum simplex* (Torr.) S. Fuentes et al., CHEHG) and many-seeded goosefoot (*Chenopodium polyspermum* L., CHEPO). Other dicotyledonous weeds were from the family of *Polygonaceae*. Of these, the most frequently occurring was lady’s thumb (*Persicaria maculosa* Gray, POLPE) which was a very problematic weed, namely in Sowing 3 (May) in 2019 when the species occurrence was massive. In the other sowing dates, its occurrence was low. Other weed species from the family of *Polygonaceae*, which occurred to a lesser extent included black bindweed (*Fallopia convolvulus* (L.) Á. Löve, POLCO) and knotgrass (*Polygonum aviculare* L., POLAV). Other less important weeds were rape (*Brassica napus* L., BRSNN), black medick (*Medicago lupulina* L., MEDLU) and Chinese mallow (*Malva verticillate* L., MALVE), which got into the stand as a crumble of preceding crops, sea mayweed (*Tripleurospermum maritimum* (L.) W. D. J. Koch subsp. Maritimum, MATIN), small-flowered Crane’s bill (*Geranium pusillum* L., GERPU), field pansy (*Viola arvensis* Murray, VIOAR), cleavers (*Galium aparine* L., GALAP), common amaranth (*Amaranthus retroflexus* L., AMARE), and night-flowering catchfly (*Silene noctiflora* L., MELNO).

The effect of the sowing date on the total number of phacelia plants and total number of weeds per m^2^ was evaluated. For the statistical assessment, the weeds were divided into groups of monocotyledonous and dicotyledonous species, and of early (winter and early spring) and late emerging species. The effect of the sowing date was evaluated on the representation of weed species in the individual groups. The statistical evaluation was made using ANOVA and the Tukey’s HSD test at a significance level of *p* < 0.05. The evaluation was focused on differences between the sowing dates in the respective years as well as on the overall effect of the sowing date and effect of the year.

The number of plants per m^2^ (Table 2) was a relatively variable indicator. Numbers of phacelia plants considerably fluctuated in the respective years, ranging from 180.7 to 318 per m^2^ at the same sowing rates. No significant influence of the sowing date was recorded on the number of phacelia plants. However, the effect of the year was observed with the highest number of phacelia plants per m^2^ being recorded in Sowing 1 in 2018 while the other sowing dates exhibited lower numbers of plants. In 2019, the highest number of phacelia plants was recorded in Sowing 2 (2nd half of April), and in 2020, it was in Sowing 3. Differences in the numbers of phacelia plants per m^2^ in the respective years were not statistically significant.

In contrast to the number of phacelia plants, the number of weeds per m^2^ was significantly affected not only by the year but also by the sowing date. The highest number of weeds was recorded in 2018. In this year, spring came very early, and the month of April was characterized by average temperatures above 10 °C and often even above 15 °C, which promoted the emergence of cockspur which is thermophilus and usually appears later in the spring. In Sowing 2 (2nd half of April), the emergence and growth of weeds were considerably suppressed due to drought, and the overall occurrence of weeds was in this year significantly lower in Sowing 2 (2nd half of April) than in the other sowing dates. In 2019, the significantly highest number of weeds was recorded in Sowing 3 (May) when the plot was massively infested by a late-spring dicotyledonous weed species, specifically by pale persicaria (*Persicaria lapathifolia* (L.) Delarbre, POLLA). In the later sowing dates, this late-spring weed species did not succeed in the closed stand canopy. The lowest total number of weeds per m^2^ was recorded in 2020 with the highest number of weeds were observed in Sowing 3 (May) again. The difference was statistically significant. Dicotyledonous and late-spring weed species slightly prevailed. Results are presented in Table 2.

The second indicator of competitiveness and stand weed infestation was the coverage of individual species per plot. The phacelia stands usually exhibited properly closed canopies. The worst stand canopy closure was recorded in Sowing 3 (May) in all years, but a significant difference was found only in 2019. Results are presented in Table 3.

### 2.5. Yield Parameters

Yield parameters, i.e., net yield of seeds per hectare, seed purity, weight of a thousand grains (WTG) and germinative capacity were evaluated after the harvest of seeds. The obtained results were assessed using analysis of variance and post-hoc Tukey’s test at a significance level *p* < 0.05. Differences between stands from sowing dates in the respective years were evaluated as well as an overall effect of the sowing date and the year. Results are presented in Table 4.

An effect (*p* < 0.05) was recorded of the sowing date on yield, seed purity, and germinative capacity. The best results were achieved in the first sowing dates when the highest yield and the best seed purity were found. The lowest yield and the worst seed purity were recorded in the third sowing dates. As to yields and seed purity, significant differences were recorded between Sowing 1 (1st half of April) and Sowing 3 (May). The influence on germinative capacity showed only in 2020, with the significantly lowest germination in Sowing 3 (May). The sowing date had no significant influence on WTG. The effect of year was significant, with the lowest yields, WTG, and germinative capacity being recorded in 2020. The lowest seed purity was recorded in 2018.

## 3. Discussion

Although our results suggested that sowing date would affect emergence of plants, differences were not statistically significant and the H0 hypothesis was not rejected. The effect of sowing date on emergence was studied also by Thrasyvoulou and Tsirakoglou [28] who evaluated sowing dates in March, June, July, and August and found that the sowing date affected the emergence. They had only a single early spring (March) sowing date in which the emergence time ranged from 8 to 11 days. A similar range of emergence time was also recorded in our experiments where we tested spring sowing dates only. In the research of Thrasyvoulou and Tsirakoglou [28], the emergence time was extended up to three times in the summer sowing dates (June and July), probably due to high temperatures. Seeds of lacy phacelia are thermosensitive [30] and in Thessaloniki where the research took place, temperatures in summer months usually rise high above 30 °C. In the conditions of the Czech Republic, the emergence of phacelia was studied by Zehnálek [31] who studied differences between varieties in summer sowing dates finding out that effect of variety on emergence is lower than effect of site. In his experiments, the emergence time was 5–15 days, which was similar to in our experiments.

The effect of sowing date on the vegetation period was studied by Bowie et al. [18], Thrasyvoulou and Tsirakoglou [28], or Lermi and Palta [29], who found out that the sowing date affects the vegetation period which was extended most significantly in the autumn sowing dates. In the case of hibernation, a total vegetation period may range even around 300 days in the autumn sowing dates; however, phacelia stands from the autumn sowing dates do not hibernate in conditions of the Czech Republic. 

The accelerated development was affected by weather conditions, namely by high temperatures combined with the lower precipitation amounts which also played a role in accelerating the development of plants from later sowing dates in our experiments. As shown in Table 5, the lowest temperatures in the period of emergence and initial development in all years were those of Sowing 1 (1st half of April) which also exhibited the longest period of vegetation from sowing to end of vegetation, which ranged from 102 to 119 days. In Sowing 2 (2nd half of April), the total vegetation period was on average 3 days shorter, reaching 100–110 days. Sowing 3 (May) exhibited the shortest period of vegetation which was on average by 8 days shorter than the vegetation period of Sowing 1 (1st half of April) and ranged from 93 to 109 days.

The height of phacelia plants is relatively variable and it is affected by numerous factors. Geren et al. [32] evaluated the effect of row width on the height of plants and found out that the height of plants is affected by the width of rows and decreases with the increasing spacing of rows. In their experiments, the height of plants ranged from 66.2 to 74.6, and was smaller than in our experiments with the narrower spacing of rows. Türk and Alagöz [33] found the height of phacelia plants increased with the increasing dose of nitrogen. Kosolapov et al. [34] studied the height of plants in various coenopopulations. The populations differed in height which ranged from 60 to 114 cm and matched the total range of heights in our experiments. The effect of sowing date on the height of phacelia plants was studied by Lermi and Palta [29] and by Bowie et al. [16] who found that the phacelia’s height is affected by the sowing date. The greatest heights were reached by plants from the autumn sowing dates, which exhibited later onset of flowering and the longest vegetation period. In later spring sowing dates, the height of plants became shorter. The onset of flowering arrived within a shorter time in later spring sowing dates than in earlier sowing dates. A similar trend was also recorded in our study with the greatest height being reached by plants from the earliest spring sowing date, which bloomed at the latest and had the longest vegetation period.

Coverage and phacelia stand canopy closing has been studied by multiple authors; however, coverage was at all times studied only in the autumn sowing dates. The research presented by us is focused on phacelia stands sown in the spring months, in which, among other things, its novelty lies. We recorded the effect of year when the worst stand canopy closure was observed in 2018 which was very dry. The best stand canopy closure was in 2020 which was colder than the preceding years and relatively moist with enough rainfall. Coverage in the autumn sowing dates of phacelia was studied for example by Brant et al. [35]. They claimed that after 60 days, an average coverage was 50%, which was markedly less than that of stands in our experiments where it ranged from 80 to 90% after 60 days. It was apparently the unfavorable impact of weather (risk of lower total precipitation amounts in spring as compared with the autumn), and they also used a sowing rate which was, by about 1/3, lower than that in our experiments. Handlířová et al. [36] inform that the coverage of phacelia from the autumn sowing dates ranged from 46 to 88% after 70 days, depending on the year. In their study, the effect of temperature and rate of its decrease during growth was observed. Higher coverage values were reached by phacelia in years with the highest October temperatures. In those years, the values of coverage approached the average values recorded in our experiments, with the measured values not demonstrating the effect of sowing rate on the number of plants per m^2^. Schappert et al. [17] observed low temperatures in October to have an unfavorable influence on stand canopy closing, too, when the coverage ranged from 60 to 80%, and a higher coverage was achieved in a warmer year.

Thompsen and Hansen [37] studied the course of canopy closure in the autumn sowing dates of phacelia and other intercrops. The coverage of phacelia was affected not only by fertilization but also by the sowing date. Phacelia from the later sowing date exhibited worse canopy closing. The course of stand canopy closure was different than in our experiments. Forty days after sowing, Sowing 1 (1st half of April) featured a closure of 23–55%, depending on fertilization. Then, the coverage increased after 53 days up to 33–65%, then the values of coverage began to fluctuate, and stagnated or slightly decreased. In the later sowing date, the canopy closure after 40 days was 11–15% depending on fertilization, and 19–31% after 54 days. The coverage gradually increased to 25–37% after 63 days from the sowing, when the observations were put to an end. Our stand canopy closure was 70–80% after 40 days and 80–90% after 50 days. The canopy closure was slower in plants from the later sowing date, and the lower coverage was most likely related to other conditions and colder weather in the autumn months. The sowing rate was lower by ½, too. A similar influence of lower temperatures on the stand closure was observed in our experiments in Sowing 1 (1st half of April) which was closing slower than stands from the other sowing dates due to colder conditions in earlier spring. Above all, the effect of sowing date, nutrition, and weather conditions on crops yield was confirmed by Kren et al. [38].

The rate of stand canopy closure has to do with competitiveness and degree of weed infestation. Dhima et al. [21] experimented with annual aromatic crops and found that phacelia had a very good competitive ability. Weed infestation of the stands of phacelia and other intercrops in Czech conditions was investigated by Brant et al. [35] who did not deal with the species composition in detail but only with the total number of weeds and their total coverage. The most significant weed in their experiments was a crumble of the preceding crop (wheat) whose total coverage was 8.8%; the coverage of other weeds altogether was 3.5%. Total mean coverage of weeds reached 12.3%, which corresponds to the overall average of weed coverage in our experiments. Unlike in the autumn sowing dates, the pre-crop crumble hardly occurred in our experiments.

The species composition of weeds in the stands of phacelia was studied by Pinke et al. [23]. In their experiments, the most significant dicotyledonous weeds with a coverage over 2% were white goosefoot (*Chenopodium album* L., CHEAL), annual ragweed (*Ambrosia artemisiifolia* L., AMBEL), knotgrass (*Polygonum aviculare* L., POLAV), and field bindweed (*Convolvulus arvensis* L., CONAR). Chenopodium album was the most significant dicotyledonous weed also in our experiments. In the experiments conducted by Pinke et al. [23], the number of monocotyledonous weeds was essentially lower than that of dicotyledonous weeds and a coverage of individual species was ca. 0.5%. The most significant monocots were yellow bristle grass (*Setaria pumila* (Poir.) Roem & Schult., SETPU), wild oat (*Avena fatua* L., AVEFA), and green bristle grass (*Setaria viridis* (L). P. Beauv., SETVI). *Setaria pumila* and *Avena fatua* occurred in our experiments, too. Weed infestation of lacy phacelia stands was also studied by Horváth and Szabó [39]. Although dicotyledonous weeds prevailed in their experiments, their species were different; the most frequently occurring ones included field bindweed (*Convolvulus arvensis* L., CONAR), tuberous pea (*Lathyrus tuberosus* L., LTHTU), and white campion (*Silene latifolia* Poir. subsp. *alba* (Mill.) Greuter & Burdet, MELAL). Dicotyledonous weeds were more represented also in experiments made by Schappert et al. [17], which were dominated by species such as shepherd’s purse (*Capsella bursa-pastoris* (L.) Medik., CAPBP), white goosefoot (*Chenopodium album* L., CHEAL), cleavers (*Galium aparine* L., GALAP), red dead-nettle (*Lamium purpureum* L., LAMPU), common chickweed (*Stellaria media* (L.) Vill., STEME), and wheat crumble. As the stands were sown in the autumn, winter species predominated, which occurred in our experiments only sporadically.

Yields and other yield parameters of lacy phacelia are affected by many factors as in the case of other agricultural crop [40]. Geren et al. [32] evaluated yields of seeds and WTG in phacelia. The yields ranged from 412 to 591 kg/ha and were affected by the row width, with wider rows showing lower yields. WTG ranged from 2.013 to 2.110 and was not affected by the row width. The yields roughly corresponded to the yields in our experiments but WTG in our experiments was lower. Kosolapov et al. [34] studied WTG in various cenopopulations. WTG differed between the populations. The detected WTG values were higher in a majority of coenopopulations than in our experiments and ranged from 1.86 to 2.44. Türk and Alagöz [33] studied the influence of nitrogen dose on the yield of seeds and WTG. Both yield and WTG increased with the increasing dose of nitrogen. Yields ranged from 130 to 209 kg/ha depending on the nitrogen dose, and the total average was lower than in our experiments. WTG ranged from 1.57 to 2.03 g according to the nitrogen dose. The average WTG in our experiments corresponded to mean values from 1.7 to 1.8 g, which more or less corresponded to the applied dose of fertilizer.

## 4. Materials and Methods

**Figure 8:**
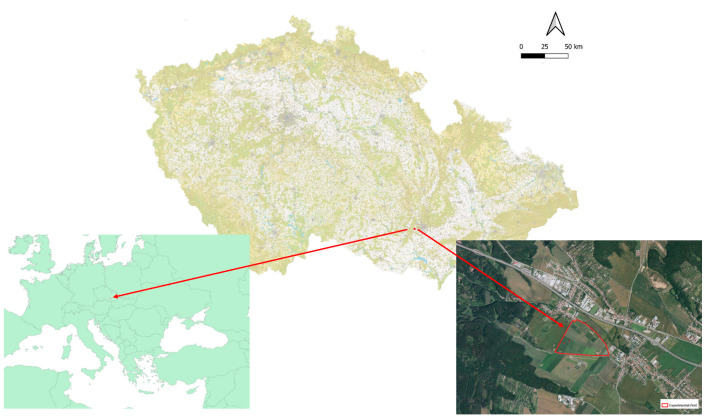
Localization of the experimental plot at Agricultural Research Ltd. In the Czech Republic and EU.

**Figure A10:**
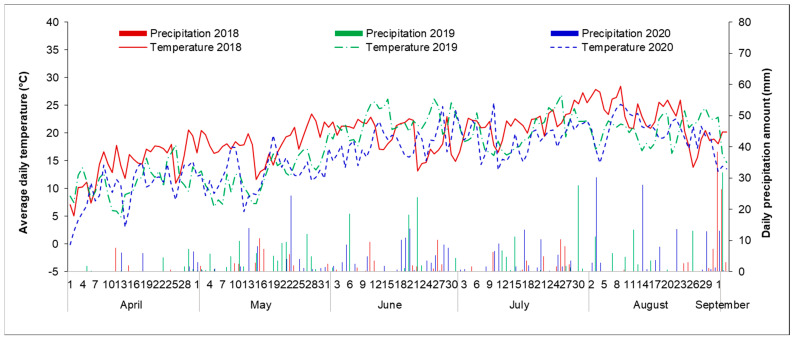
Average daily temperatures and daily precipitation amounts in the growing seasons of period 2018–2020. The values were measured by the professional meteorological station (ID: B2TROU01; type: AKS2) which is operated by the Czech Hydrometeorological Institute.

In 2018–2020, small-plot experiments were established in the cadastral area of Troubsko village (Figure 8) at an altitude of 270 m a.s.l. on the degraded chernozem with the neutral pH reaction of 6.4 and humus content of 2%. An average content of nutrients in arable soil was 216 mg K, 91 mg P, and 79 mg N. Basic fertilization with 300 kg/ha N-P-K (10-26-26) (HOKR, spol. S r.o., Pardubice, Czech Republic) was applied before sowing. There was no chemical protection. The course of weather during the growing season of lacy phacelia for the respective years and sowing dates is presented in Table 5 and Table 6. A comparison of weather course in the respective years is shown in Appendix B, Figure A10.

**Figure A11:**
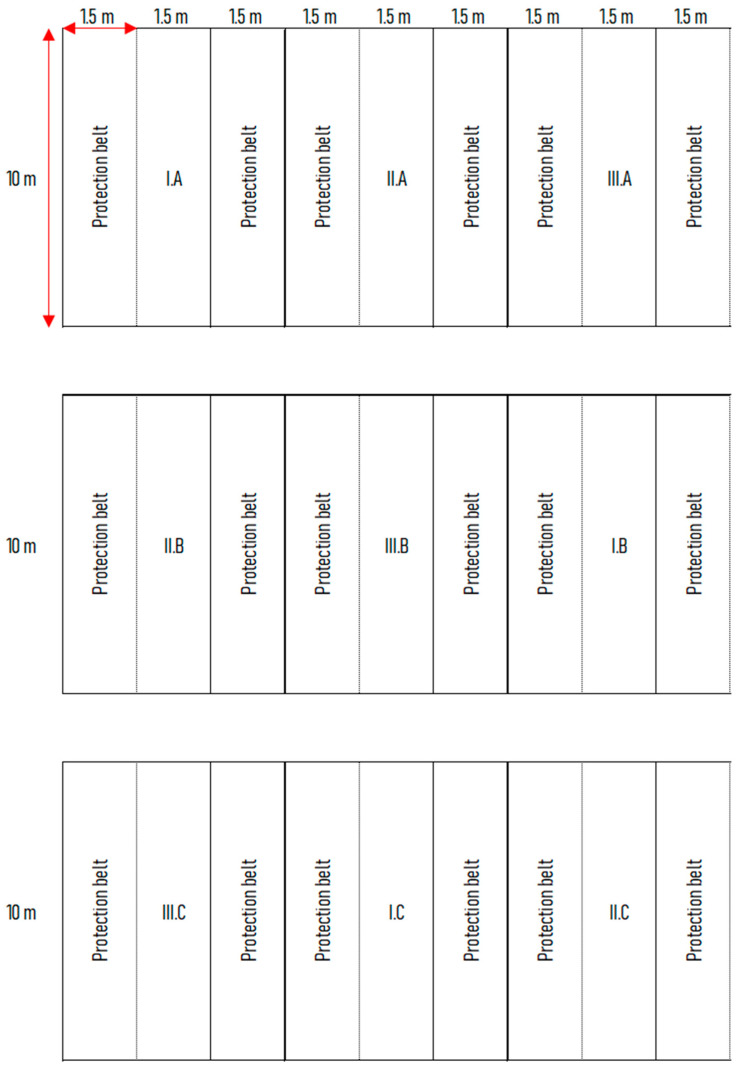
Organization of the field experiment: I (A,B,C)—Sowing 1 (1st half of April); II (A,B,C)—Sowing 2 (2nd half of April); III (A,B,C)—Sowing 3 (May).

Experiments were carried out in triplicates and the size of experimental plots was 15 m^2^, i.e., 1.5 × 10 m every year (2018, 2019, and 2020) An overview of the organization of the field experiment is shown in Appendix B, Figure A11. One experimental plot represented one repetition of one specific variant. In total, three variants of the experiment were prepared with different dates of sowing (1st term—the first half of April; 2nd term—the second half of April; and 3rd term—May; Table 5). A 1.5 m wide control strip was created around each experimental area. The above protection belts of 1.5 m in width were established to eliminate the influence of margins on each plot. Sowing by means of no-residue Oyord seeding machine was implemented on three dates: in the first half of April, in the second half of April, and in the first half of May (see Table 5). Time gaps between the respective sowing dates were ca. 14 days. Row width was 12.5 cm. In order to eliminate the effect of variety and sowing rate, seeds of the same variety (Czech variety Meva) were used in all years as well as the same sowing rate of 7 million individuals per hectare, recommended for rows of 12.5 cm, which is 5–8 million individuals per hectare [41].

During the growing season, phenological stages of lacy phacelia were recorded. The phenological stages were evaluated according to the general BBCH scale [42], which was modified for the assessment of lacy phacelia [43]. The most important developmental stages that were recorded included BBCH 00 (sowing), BBCH 10 (emergence), BBCH 12 (first true leaves), BBCH 16 (development of true leaves, BBCH 31 (beginning of stem elongation), BBCH 51 (beginning of bud formation), BBCH 55 (butonization—bud formation), BBCH 61 (beginning of flowering), BBCH 65 (full bloom), BBCH 72 (beginning of fruit formation), BBCH 89 (ripening), BBCH 98 (end of vegetation). The beginning of individual stages was considered to be when the particular stage was reached by min. 10% of plants.

Dates when the stand reached the given developmental stages were recorded. Up to BBCH 98, the height of plants was assessed according to EPPO 1/189 (2) methodology. The coverage of phacelia in % per plot was evaluated until the beginning of seed formation, i.e., up to BBCH 72. In the later stages, the stand was often lodging, and the coverage of intact stand could not be evaluated objectively.

Daily height increments for individual time periods between the subsequent developmental stages were calculated from the detected height values and time difference between reaching the growth stages according to the below Formula (1):

DPV = (V_n_ − V_n−1_)/(t_n_ − t_n−1_), (1)

where DPV = daily height increment, V_1_ = height at t_n_, V_0_ = height at t_n−1_, t_n_ = time for reaching the next growth stage, t_n−1_ = time of reaching the preceding developmental stage; and growth intensity was recorded in individual intervals between the studied growth stages.

Daily coverage increments for individual time periods between the subsequent developmental stages were calculated from the detected coverage values and time difference between reaching the respective growth stages according to the below Formula (2):

DPP = (P_n_ − P_n−1_)/(t_n_ − t_n−1_), (2)

where DPP = daily coverage increment, P_n_ = coverage at t_n_, P_n−1_ = coverage at t_n−1_, t_n_ = time of reaching the next growth stage, t_n−1_ = time of reaching the preceding developmental stage; and intensity of stand canopy closure was recorded in individual intervals between the studied growth stages. The formulas were obtained by modifying the formula for calculating the periodic annual increment (PAI) according to Ábri et al. [44].

At the beginning of flowering (BBCH 61), the competitiveness of phacelia was evaluated when not only its coverage was recorded but also the coverage of weeds in % per plot, and the number of plants was counted both for the lacy phacelia and for individual weed species. For counting the plants, a template was used sized 1/4 m. Two counts were made per plot and results were converted to 1 m^2^. Stand ripening was followed by harvesting seeds with the use of Sampo small plot combine harvester (Sampo Rosenlew, Pori, Finland), and yield parameters were assessed (yield, weight of a thousand grains, seed purity and germinative capacity). Net plots without protective belts were harvested.

The measured values of respective parameters were analyzed using the STATISTICA 12 program (Dell, Round Rock, TX, USA). Average values of individual parameters, dispersal of measured values, and significance of differences in the average values of individual parameters were determined using ANOVA and post-hoc Tukey’s HSD test. All analyses were made at a significance level of *p* < 0.05.

## 5. Conclusions

In our field experiment, the effect of sowing date on the growth and overall condition of phacelia seed stands was demonstrated. The generally best results were achieved in the first sowing dates whereas the latest third sowing dates appeared the least effective. In later sowing dates, the height of plants was decreased depending on weather and year, and the stands exhibited worse canopy closure, especially in 2019 when a significant difference was recorded in the canopy closure of the latest (third) sowing date. The third sowing date also showed a higher susceptibility of stands to weed infestation. The worse condition of the stand affected yield parameters, i.e., yield of seeds and seed purity. The effect of sowing date on the weight of a thousand of grains was not unambiguous and significant differences were not observed. In germinative capacity, significant differences were recorded only in one year in which the germinative capacity of plants from the later sowing dates was reduced.

## Figures and Tables

**Table 1:** Analysis of variance for number of days, plant height, coverage, and daily height and coverage increment.

Characteristic	df	Days	Height	df	Coverage	df	Days between Stages	Daily Height Increment	df	Daily Coverage Increment
Year (Y)	2	***	***	2	***	2	***	***	2	*
Sowing (S)	2	***	***	2	***	2	***	***	2	**
Stage (ST)	11	***	***	7	***	10	***	***	6	***
YxS	4	NS	***	4	**	4	***	***	4	**
YxST	22	NS	***	14	*	20	NS	***	12	**
SxST	22	NS	***	14	NS	20	NS	***	12	**
YxSxST	44	-	***	28	NS	40	-	***	24	**
	df	*p*-value	*p*-value	df	*p*-value	df	*p*-value	*p*-value	df	*p*-value
Year (Y)	2	<0.001	<0.001	2	<0.001	2	<0.001	<0.001	2	0.019
Sowing (S)	2	<0.001	<0.001	2	<0.001	2	<0.001	<0.001	2	0.003
Stage (ST)	11	<0.001	<0.001	7	<0.001	10	<0.001	<0.001	6	<0.001
YxS	4	0.992	<0.001	4	0.001	4	<0.001	<0.001	4	0.001
YxST	22	0.363	<0.001	14	0.044	20	0.635	<0.001	12	<0.001
SxST	22	0.990	<0.001	14	0.100	20	0.998	<0.001	12	<0.001
YxSxST	44	-	<0.001	28	0.942	40	-	<0.001	24	<0.001

Legend: NS—non-significant; *—significant level at the 0.05 (*p* < 0.05); ** significant level at 0.01 (*p* < 0.01); *** significant level at 0.001 (*p* < 0.001); Days—number of days for reaching the respective development stages; Days between stages—number of days for reaching the range of developmental stages.

**Table 2:** Effect of sowing date on number of plants per m^2^.

Year	Sowing	Lacy Phacelia	Weeds Total	Weeds Monocots	Weeds Dicots	Weeds Winter and Early Spring	Weeds Late Spring
2018	1	318.0 ^a^	210.0 ^a^	111.3 ^a^	98.7 ^ab^	98.7 ^ab^	111.3 ^a^
2018	2	266.0 ^a^	38.7 ^b^	15.3 ^b^	23.3 ^b^	22.0 ^b^	16.7 ^b^
2018	3	216.0 ^a^	182.7 ^a^	63.3 ^ab^	119.3 ^a^	114.7 ^a^	68.0 ^a^
2019	1	207.3 ^a^	23.3 ^b^	8.0 ^a^	15.3 ^b^	16.7 ^a^	6.7 ^b^
2019	2	293.3 ^a^	28.7 ^b^	2.0 ^a^	26.7 ^b^	20.7 ^a^	8.0 ^b^
2019	3	210.0 ^a^	342.7 ^a^	9.3 ^a^	333.3 ^a^	34.7 ^a^	308.0 ^a^
2020	1	224.0 ^a^	28.7 ^b^	13.3 ^b^	15.3 ^b^	13.3 ^ab^	15.3 ^b^
2020	2	180.7 ^a^	22.7 ^b^	8.7 ^b^	14.0 ^b^	11.3 ^b^	11.3 ^b^
2020	3	228.7 ^a^	61.3 ^a^	23.3 ^a^	38.0 ^a^	23.3 ^a^	38.0 ^a^
**Average for individual sowing dates**							
	1	249.8 ^a^	87.3 ^b^	44.2 ^a^	43.1 ^b^	42.9 ^ab^	44.4 ^b^
	2	246.7 ^a^	30.0 ^b^	8.7 ^b^	21.3 ^b^	18.0 ^b^	12.0 ^b^
	3	218.2 ^a^	195.6 ^a^	32.0 ^a^	163.6 ^a^	57.6 ^a^	138.0 ^a^
**Average for individual years**							
2018		266.7 ^a^	143.8 ^a^	63.3 ^a^	80.4 ^ab^	78.4 ^a^	65.3 ^ab^
2019		236.9 ^ab^	131.6 ^a^	6.4 ^b^	125.1 ^a^	24.0 ^b^	107.6 ^a^
2020		211.1 ^b^	37.6 ^b^	15.1 ^b^	22.4 ^b^	16.0 ^b^	21.6 ^b^

Legend: Average values (*n* = 6) are shown of the number of plants in the respective experimental years, in the respective variants and totals of all variants for the specific years (*n* = 54). Different letter indices illustrate statistical differences at a level of *p* < 0.05 (ANOVA, post-hoc Tukey’s HSD test). Sowing: 1—1st half of April; 2—2nd half of April; 3—May.

**Table 3:** Effect of sowing date on coverage of plants on the plot.

Year	Sowing	Lacy Phacelia	Weeds Total	Weeds Monocots	Weeds Dicots	Weeds Winter and Early Spring	Weeds Late Spring
2018	1	91.7 ^a^	15.4 ^a^	6.0 ^a^	9.3 ^a^	9.3 ^a^	6.1 ^a^
2018	2	86.7 ^a^	9.6 ^a^	5.4 ^a^	4.2 ^a^	3.7 ^a^	5.9 ^a^
2018	3	79.7 ^a^	32.1 ^a^	15.7 ^a^	16.4 ^a^	16.0 ^a^	16.1 ^a^
2019	1	92.5 ^a^	10.1 ^a^	4.1 ^a^	6.0 ^a^	7.8 ^a^	2.3 ^a^
2019	2	98.7 ^a^	2.4 ^a^	0.1 ^b^	2.3 ^a^	1.4 ^b^	1.0 ^a^
2019	3	80.0 ^b^	22.2 ^a^	0.8 ^b^	21.4 ^a^	1.1 ^b^	21.1 ^a^
2020	1	97.3 ^a^	4.0 ^b^	2.2 ^a^	1.8 ^b^	1.8 ^a^	2.3 ^b^
2020	2	98.3 ^a^	3.3 ^b^	1.5 ^a^	1.8 ^b^	1.6 ^a^	1.8 ^b^
2020	3	97.0 ^a^	12.8 ^a^	4.3 ^a^	8.5 ^a^	4.7 ^a^	8.1 ^a^
**Average for in-dividual sowing dates**							
	1	93.6 ^a^	8.9 ^b^	3.7 ^ab^	5.2 ^b^	5.6 ^a^	3.4 ^b^
	2	94.6 ^a^	5.1 ^b^	2.4 ^b^	2.8 ^b^	2.2 ^a^	2.9 ^b^
	3	85.6 ^a^	22.4 ^a^	6.9 ^a^	15.4 ^a^	7.3 ^a^	15.1 ^a^
**Average for individual years**							
2018		86.0 ^b^	19.0 ^a^	9.0 ^a^	10.0 ^a^	9.7 ^a^	9.4 ^a^
2019		90.1 ^ab^	10.7 ^ab^	1.3 ^b^	9.4 ^a^	2.7 ^b^	8.0 ^a^
2020		97.6 ^a^	6.7 ^b^	2.7 ^b^	4.0 ^a^	2.7 ^b^	4.0 ^a^

Legend: Average values (*n* = 3) are shown of % coverage of soil in the respective experimental years, in the respective variants and totals of all variants for the specific years (*n* = 27). Different letter indices illustrate statistical differences at a level of *p* < 0.05 (ANOVA, post-hoc Tukey´s HSD test). Sowing: 1—1st half of April; 2—2nd half of April; 3—May.

**Table 4:** Effect of sowing date on yield parameters.

Year	Sowing	Yield kg/10,000 m^2^	Purity	WTG	Germination
2018	1	496.3 ^a^	92.4 ^a^	1.9867 ^a^	99.0 ^a^
2018	2	422.4 ^a^	86.8 ^a^	1.9450 ^a^	97.7 ^a^
2018	3	429.1 ^a^	64.9 ^b^	1.7217 ^b^	98.3 ^a^
2019	1	571.5 ^a^	90.8 ^a^	1.6400 ^a^	98.7 ^a^
2019	2	348.3 ^b^	81.5 ^a^	1.8167 ^a^	99.3 ^a^
2019	3	277.6 ^b^	87.0 ^a^	1.8933 ^a^	99.3 ^a^
2020	1	230.4 ^a^	96.8 ^a^	1.7076 ^a^	92.7 ^b^
2020	2	182.7 ^a^	97.1 ^a^	1.7117 ^a^	95.1 ^a^
2020	3	52.0 ^b^	92.9 ^b^	1.6314 ^a^	86.0 ^c^
**Average for in-dividual sowing dates**					
	1	432.7 ^a^	93.3 ^a^	1.7781 ^a^	96.8 ^a^
	2	317.8 ^b^	88.4 ^ab^	1.8245 ^a^	97.4 ^a^
	3	252.9 ^b^	81.6 ^b^	1.7488 ^a^	94.6 ^b^
**Average for individual years**					
2018		449.2 ^a^	81.3 ^a^	1.8844 ^a^	98.3 ^a^
2019		399.1 ^a^	86.4 ^ab^	1.7833 ^a^	99.1 ^a^
2020		155.0 ^b^	95.6 ^b^	1.6836 ^b^	91.3 ^b^

Legend: Average values (*n* = 3) are shown of the phacelia yields in the respective experimental years, in the respective variants and totals of all variants for the specific years (*n* = 27). Different letter indices illustrate statistical differences at a level of *p* < 0.05 (ANOVA, post-hoc Tukey´s HSD test). Sowing: 1—1st half of April; 2—2nd half of April; 3—May.

**Table 5:** 10-day temperature averages in °C for individual sowing dates.

Sowing Date	D0	D10	D20	D30	D40	D50	D60	D70	D80	D90	D100	D110	D120
12 April 2018	11.2	15.6	16.5	18.0	15.9	20.5	21.3	20.3	17.2	20.0	20.9	23.1	26.3
24 April 2018	15.8	17.0	17.7	16.0	20.9	21.4	19.4	16.8	20.4	21.7	24.1	25.4	23.4
10 May 2018	18.0	16.1	19.8	21.1	20.2	17.8	19.9	20.4	22.5	26.0	23.2	21.7	19.1
05 April 2019	9.1	8.8	12.2	12.6	9.6	12.7	16.9	21.7	22.0	22.6	18.2	20.0	22.9
16 April 2019	8.6	12.9	11.6	9.6	13.6	17.4	22.1	21.8	22.2	17.9	20.7	22.2	20.7
07 May 2019	10.6	9.8	14.3	17.8	22.5	21.6	21.8	17.8	21.5	21.5	20.6	20.3	21.1
08 April 2020	4.5	10.1	11.5	12.0	11.7	14.7	14.6	18.4	17.6	20.9	17.2	18.8	19.9
22 April 2020	10.5	12.3	12.6	12.1	13.1	16.3	18.7	19.0	20.3	16.8	20.1	20.6	21.7
07 May 2020	11.9	11.6	14.5	14.3	18.3	17.7	20.6	17.7	18.5	20.1	22.6	20.6	17.3
Average Sowing 1	8.2	11.5	13.4	14.2	12.4	16.0	17.6	20.1	19.0	21.2	18.8	20.6	23.0
Average Sowing 2	11.7	14.1	14.0	12.6	15.8	18.4	20.1	19.2	21.0	18.8	21.6	22.8	21.9
Average Sowing 3	13.5	12.5	16.2	17.7	20.3	19.0	20.8	18.6	20.8	22.5	22.1	20.9	19.2
Overall average	11.1	12.7	14.5	14.8	16.2	17.8	19.5	19.3	20.3	20.8	20.8	21.4	21.4

Legend: D0 = Days 1–10 before sowing, D10-D120 = Decades 1–12 after sowing. The average temperatures are always shown for 10 days and divided by decades: 10 days from sowing (D10); 20 days from sowing (D20) etc.

**Table 6:** Precipitation amounts (mm) in 10 days for individual sowing dates.

Sowing Date	D0	D10	D20	D30	D40	D50	D60	D70	D80	D90	D100	D110	D120
12 April 2018	0.0	9.8	0.9	7.1	21.0	7.8	4.8	13.3	17.4	7.9	4.2	27.6	0.0
24 April 2018	2.2	2.6	5.2	26.6	4.7	15.5	3.9	13.6	8.0	9.2	22.5	0.7	0.0
10 May 2018	2.0	26.2	7.8	4.5	13.6	17.4	1.6	10.5	27.6	0.0	0.7	5.8	79.0
05 April 2019	8.6	2.4	4.5	17.3	19.0	33.9	19.3	18.7	45.3	4.4	11.7	12.3	32.0
16 April 2019	9.8	4.5	18.2	24.1	27.9	19.3	18.7	45.3	4.4	12.2	11.8	43.3	26.7
07 May 2019	2.0	24.3	27.7	19.3	18.7	45.3	4.4	23.5	0.5	43.3	26.8	4.3	45.2
08 April 2020	8.5	6.1	6.0	12.7	23.8	33.7	16.9	9.7	47.5	17.9	19.3	34.7	39.5
22 April 2020	9.8	11.2	3.1	22.2	37.1	21.2	37.5	28.5	9.6	28.0	21.4	35.9	40.5
07 May 2020	2.0	23.8	33.7	16.9	9.7	42.3	23.1	19.0	33.3	41.2	27.9	26.2	29.5
Average Sowing 1	5.7	6.1	3.8	12.4	21.3	25.1	13.7	13.9	36.7	10.1	11.7	24.9	23.8
Average Sowing 2	7.3	6.1	8.8	24.3	23.2	18.7	20.0	29.1	7.3	16.5	18.6	26.6	22.4
Average Sowing 3	2.0	24.8	23.1	13.6	14.0	35.0	9.7	17.7	20.5	28.2	18.5	12.1	51.2
Average	5.0	12.3	11.9	16.7	19.5	26.3	14.5	20.2	21.5	18.2	16.3	21.2	32.5

Legend: D0 = Days 1–10 before sowing, D10–D120—Decades 1–12 after sowing. The precipitation amounts are always shown for 10 days and divided by decades. 10 days from sowing (D10); 20 days from sowing (D20) etc.

## Data Availability

All data used are available from the authors.

## Appendix A

Number of plants and leaf coverage (2018–2020).

## Appendix B

Weather conditions.
